# The Effect of Mismatch Repair - Deficient/ Microsatellite Instability on Black Minority Colorectal Cancer Gene Expression

**DOI:** 10.51894/001c.143853

**Published:** 2025-10-01

**Authors:** Dimitri F. Joseph, Joseph F. LaComb, Andrew Fu, Ricardo E. Flores, Ellen Li, Alexandra Guillaume, Bin Chen

**Affiliations:** 1 College of Osteopathic Medicine Michigan State University

## 11

### BACKGROUND

Compared to all other racial groups, black Americans are most likely to be diagnosed with colorectal cancer (CRC) and are most likely to die from the disease. These racial disparities have been largely attributed to social and economic conditions that impact the prevalence of co-morbidities and access to quality health care. However, the molecular and cellular differences in CRC tumors between black and white subjects remain largely uncharacterized. In this study, we evaluate mismatch repair status (MMR) as a confounder to immune related differences between racial groups. We demonstrate that the difference in CXCL10 expression across black and white American CRC samples may be driven by a disproportionate prevalence of mismatch repair status / microsatellite instability.

### METHODS

We analyzed bulk RNA sequencing collected from two independent colorectal cancer datasets. RNA-seq expression values for TCGA samples were dowloaded from OCTAD while clinical annotations for matching samples were extracted from cross-referencing Genomic Data commons and . Samples from Greater Poland and Brazil were excluded from analysis. Differential expression analysis was EdgeR and the Wilcoxon rank sum tests from the OCTAD package. Significant genes were determined by an absolute log 2-fold change > 1, adjust p value < 0.05. Pathway analysis using Gene set enrichment analysis (GSEA) of hallmark gene sets was applied to the TCGA gene list ranked by fold changes. Gene Ontology (GO) enrichment analysis of significant DEGs was performed using enrichR.

T- test was performed for MSI across racial groups.

Log2 normalized CXCL10 expression values was evaluated for 273 TCGA colorecatal cancer subjects in a general linear model fitting a negative binomial distribution. TCGA annotations from the Covariables Univariate analysis The general linear model compared covariates

This research first identifies that CXCL10 is significantly downregulated in Black colorectal cancers in two independent datatsets. We also observe that immune related pathways and cells are significantly different between the self identified Black and White Americans from The Cancer Genome Atlas. We use a combination of RT-qPCR, bulk RNA seq anal to show that CXCL10 expression is higher in the MMRdeficient CRC subtype.

